# Editorial Comment on “Efficacy of pembrolizumab plus lenvatinib as first‐line treatment for metastatic renal cell carcinoma with multiple brain metastases”

**DOI:** 10.1002/iju5.12805

**Published:** 2024-11-12

**Authors:** Manabu Kato

**Affiliations:** ^1^ Department of Urology Aichi Cancer Center Hospital Nagoya Aichi Japan

Matsumoto *et al*. reported a case of renal cell carcinoma (RCC) with multiple brain metastases (BMs) treated with stereotactic brain radiation followed by pembrolizumab plus lenvatinib.^1^ As demonstrated in the Discussion section of this case report, monotherapies with tyrosine kinase inhibitors (TKI), such as cabozantinib, or immune checkpoint inhibitors have exhibited efficacy in improving survival outcomes in the management of BMs from RCC. Therefore, proactive drug treatment for BMs from RCC is warranted. In this regard, Takemura et al. recently reported the outcomes of 389 patients with BMs from RCC utilizing data from the International Metastatic Renal Cell Carcinoma Database Consortium.^2^ In this study, a significant difference in overall survival (OS) was observed between patients with BMs from RCC receiving IO‐based combination as first‐line drug therapy (32.7 months) compared with those receiving TKI monotherapy (20.6 months). Meanwhile, the group treated with stereotactic radiation or neurosurgery for multiple BMs from RCC showed a longer OS of 31.4 months compared with the group treated with whole brain radiation or no radiation (16.5 months). Yomoet al. reported a longer survival without increase in adverse event after stereotactic brain radiation with IO combination therapy for BMs from RCC.^3^

Thus far, stereotactic radiation, monotherapy with TKI, or IO have shown effectiveness in controlling BMs in patients with RCC. With more evidence from the aforementioned articles, multimodality treatments composed of stereotactic radiation and IO plus TKI could improve the OS of patients with RCC with multiple BMs.

## Author contributions

Manabu Kato: Writing – review and editing.

## Conflict of interest

The authors declare no conflict of interest.
